# Global research trends on innate lymphoid cells in the brain, gut and lung field: a bibliometric and visualized analysis

**DOI:** 10.3389/fimmu.2024.1336666

**Published:** 2024-02-07

**Authors:** Jianliang Huang, Kun Deng, Ying Liu, Mingkai Xia, Mingsheng Lei, Minghua Wu

**Affiliations:** ^1^ Zhangjiajie Hospital Affiliated to Hunan Normal University, Zhangjiajie, China; ^2^ The Key Laboratory of Carcinogenesis of the National Health Commission, The Key Laboratory of Carcinogenesis and Cancer Invasion of the Chinese Ministry of Education, Cancer Research Institute, Central South University, Changsha, Hunan, China; ^3^ Medical College of Jishou University, Jishou, China; ^4^ Zhangjiajie College, Zhangjiajie, China

**Keywords:** bibliometric analysis, ILCs, brain-gut-lung axis, CiteSpace, VOSviewer, HistCite

## Abstract

**Background:**

ILCs play important roles in the brain, gut, and lungs. Researchers are attempting to establish a research framework on the brain-gut-lung axis using ILCs. However, no one has yet conducted a bibliometric analysis to summarize the findings. In this study, we utilized bibliometrics to analyze the emerging trends and focal areas of ILCs in the brain, intestine, and lung. We aim to provide references for future research on the brain-gut-lung axis.

**Methods:**

To conduct a comprehensive bibliometric analysis on ILCs in the fields of brain, intestine, and lung, we utilized software such as HistCite, VOSviewer, and CiteSpace. Our analysis focused on various aspects, including the number of publications, countries, authors, journals, co-cited documents, and keywords. This approach allowed us to gain valuable insights into the research landscape surrounding ILCs in these specific fields.

**Results:**

A total of 8411 articles or reviews on ILCs in the fields of brain, intestine, and lung were included. The number of published articles has shown a consistent upward trend since 2003. A total of 45279 authors from 99 countries have contributed to these articles. The United States has the highest number of publications (n=3044) and the most cited articles (TGCS=210776). The top three published authors in this field are David Artis, Marco Colonna and Andrew NJ McKenzie. The journal Immunity is the most authoritative choice for researchers. The main research focuses in this field include NK cell, ILC2, tumor immunity, multiple sclerosis, inflammatory bowel disease, airway inflammation, RORγT, and immunotherapy. In recent years, cancer and tumor microenvironment have emerged as hot keywords, particularly immunotherapy, PD-1 related directions, indicating a potential shift in research focus.

**Conclusion:**

European and American countries have been pivotal in conducting research on ILCs, while China has produced a significant number of publications, its impact is still limited. Tumors are likely to emerge as the next focal points in this field. The connection and regulation between the brain and the lung are not yet fully understood, and further investigation is necessary to explore the role of ILCs in the brain-lung axis.

## 1 Introduction

Innate lymphoid cells (ILCs) are a type of immune cells that lack adaptive antigen receptors. Similar to T and B lymphocytes, they originate from common lymphoid progenitor cells and can be rapidly activated during the early stages of disease to provide immunoprotective effects. These cells are widely distributed in mucosal barriers such as the lungs, brain, intestines, and skin. They play a crucial role in the body’s immune defense, tissue repair, inflammatory response, metabolic homeostasis, and tumor suppression (1, 2). According to the naming rules established by Spits et al., ILCs can be categorized into three major groups (3). Group 1 consists of NK cells and ILC1, which rely on T-box transcription factors to produce IFN-γ and primarily respond to viruses and tumors. Group 2 comprises ILC2 that produce IL-5 and IL-13. These cells mainly rely on the transcription factors GATA3 and RORA and respond to parasites and allergens. The final group, Group 3, includes LTi and ILC3 that produce IL-17 and IL-22. These cells primarily act on pathogenic microorganisms such as bacteria and fungi (4). Consequently, ILCs have garnered significant attention in various medical fields, becoming a prominent area of research.

The connection and regulation between the brain, intestines, and lungs are still not fully understood in detail. Recent research has emphasized the important role of ILCs. According to Quatrini and colleagues, ILC cells have a high expression of catecholamine and glucocorticoid receptors on their surface. These receptors are regulated by the hypothalamus-pituitary-adrenergic axis, sympathetic nerves, and parasympathetic nerves (4, 5). Cardoso et al. found that sympathetic nerves can regulate the activity of ILC2 in adipose tissue and activate ILC2 cells through β2AR (6). Furthermore, IL-33 can enhance the activation of ILC2, promoting macrophage activation and eosinophil aggregation to regulate insulin resistance and obesity (7, 8). Catecholamines have been found to negatively regulate the immune response of ILC2, thereby reducing type 2 inflammatory responses in lung and intestinal tissues (9).In the research conducted by von Moltke and Mjösberg (10, 11), it has been observed that helminth infection or IL-25 stimulation can lead to the activation and proliferation of a large number of ILC2s in the intestines. This activation results in the release of type 2 cytokines (IL-4, IL-5, IL-13) and the promotion of immune cell activation (such as mast cells and eosinophils). Through the mediation of sphingosine 1-phosphate (S1P), ILC2s can migrate through the blood circulation to other parts of the body (12), including the lungs. This migration contributes to both anti-worm defense and tissue repair. However, it also leads to the production of inflammatory ILC2s in the lungs, which can cause allergic diseases and support Mjösberg’s hypothesis that lung inflammation originates from the intestines (11). Another study by *Cao* et al. discovered that dopamine can inhibit the generation of ILC2s and type 2 inflammatory responses by regulating the mitochondrial oxidative phosphorylation process, thereby preventing the occurrence of asthma (13). Therefore, conducting in-depth research on the mechanism of action of ILCs will enhance our understanding of the correlation and regulatory mechanisms between the brain, intestines, and lungs.

Bibliometrics is a research method that focuses on analyzing the literature system and related media to identify research hotspots and development trends in various fields (14, 15). It combines mathematical and statistical methods with visual analysis to provide valuable insights for academic research and innovation. However, our search on platforms like Web of Science, PubMed, and China National Knowledge Infrastructure did not yield any bibliometric or visual analysis on ILCs related to the brain, intestines, and lungs. Therefore, This article utilizes bibliometric methods to comprehensively evaluate the research trends, hotspots, and current status of ILCs in the brain, intestine, and lung domains during the last two decades. It aims to bridge the gap in bibliometric studies in this specific area and provides valuable insights for future research on the brain-gut-lung axis.

## 2 Materials and methods

### 2.1 Data retrieval and collection

**Figure 1:**
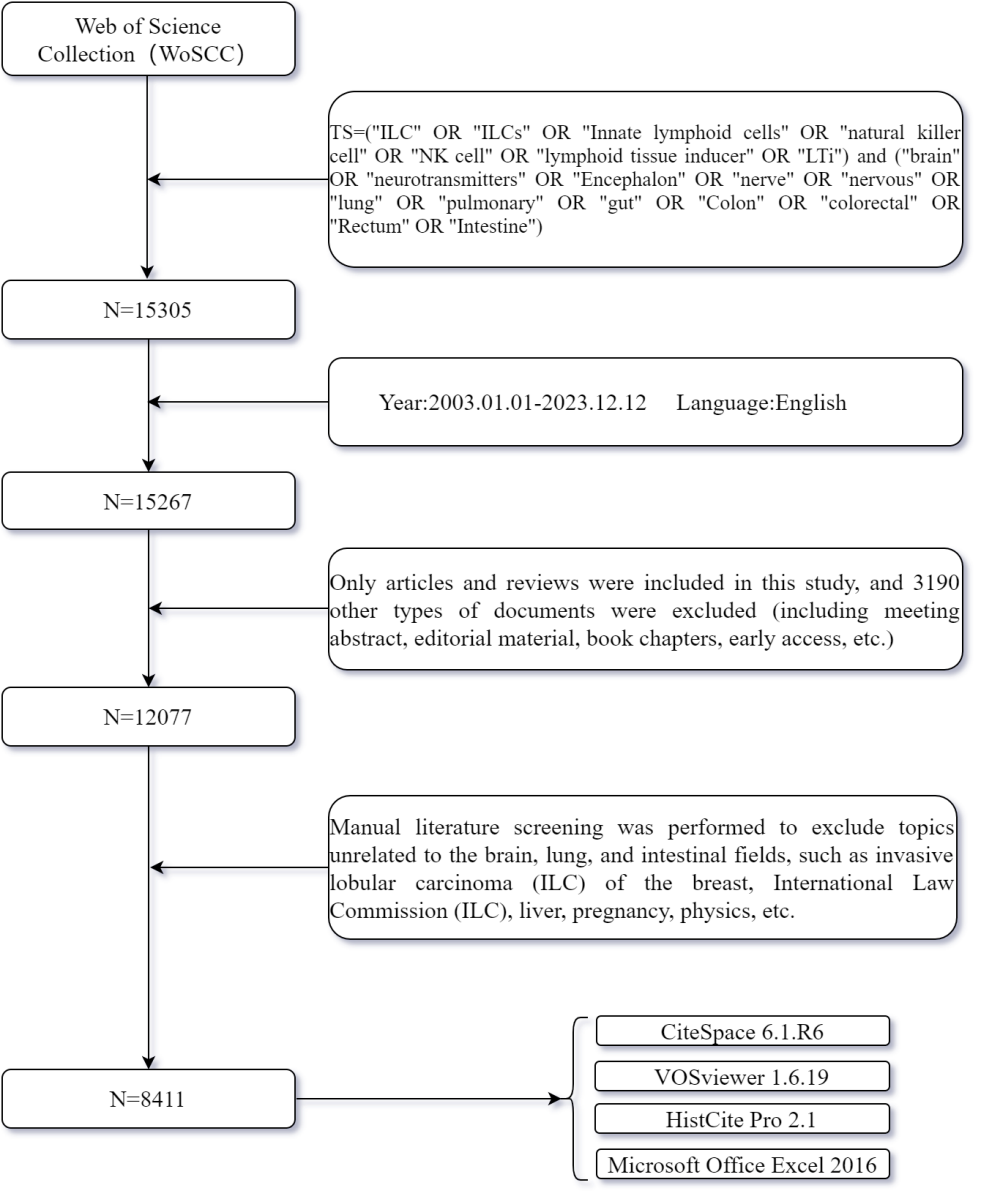
Flow chart of data collection in this study.

We searched the Web of Science Core Collection (WoSCC) to find articles on ILCs in the brain, intestine, and lung fields over the past 20 years (from 2003-01-01 to 2023-12-12). The search formula used was TS=(“ILC” OR “ILCs” OR “Innate lymphoid cells” OR “natural killer cell” OR “NK cell” OR “lymphoid tissue inducer” OR “LTi”) and (“brain” OR “neurotransmitters” OR “Encephalon” OR “nerve” OR “nervous” OR “lung” OR “pulmonary” OR “gut” OR “Colon” OR “colorectal” OR “Rectum” OR “Intestine”). The search was completed on December 12, 2023, to ensure data accuracy. Articles written in English were specifically chosen, and the document types were limited to articles and reviews, excluding meeting abstracts, editorial materials, proceedings papers, and other document types. Manual literature screening was conducted to exclude irrelevant articles, such as those related to testicular interstitial cells (ILC), breast invasive lobular carcinoma (ILC), International Law Commission (ILC), etc. Additionally, content that was unrelated to the fields of brain, lung, and intestine, such as liver, uterus, pregnancy, blood system diseases, breast diseases and physical realm, was also excluded (**Figure 1**). The search results were then exported in Plain Text file format, and the Full Record and Cited References of the documents were collected. This included information such as authors, countries, institutions, references, keywords, journals, and other relevant details. These data were utilized for bibliometric and visual analysis purposes.

### 2.2 Data analysis and visualization

In this study, we will utilize HistCite, VOSviewer, and CiteSpace for bibliometric and visual analysis. Additionally, we will employ Microsoft Excel Office to organize relevant data including the number of publications and citation frequency.

Histcite is a document analysis tool developed by Thomson Reuters in the United States (16). It is specifically designed to analyze documents (Plain Text files) exported from the WoS database. The tool effectively identifies and counts various elements such as keywords, authors, countries, institutions, journals, publication years, languages, document types, cited documents, TLCS, TGCS, and other relevant information in each document. Additionally, it constructs the citation relationships between documents, which aids in understanding the overall context and relationships within a particular field. TLCS, also known as the total local citation score, indicates the level of attention a document has received from peers. A higher TLCS score implies greater attention and makes the document more worthy of reading. On the other hand, TGCS, or the total global citation score, takes into account the attention the document has received from other fields or disciplines.

VOSviewer is a powerful document visualization tool developed by Leiden University. It facilitates multi-dimensional bibliometric and visual analysis (17), including network visualization, coverage visualization, and density visualization. Through the construction of network relationship maps between authors, keywords, citations, etc., it aids in uncovering cooperative relationships, research hotspots, and development trends in the field. Network visualization depicts connections between documents, keywords, and authors using a network diagram. The size of nodes represents the frequency of occurrence of the corresponding unit, and clusters are indicated by the same color. Overlay visualization adds supplementary information to the original network diagram, providing further insights. Density visualization illustrates the distribution of a specific attribute in the network diagram, enabling the visualization of heat distribution.

Citespace, a document visualization software developed by Professor Chen, is capable of analyzing massive documents from various databases such as Web of Science, Pubmed, and CNKI. It provides insights on topics, keywords, author units, collaborative networks, journals, publication time, and document citations (18). By performing spatiotemporal analysis, hot spot analysis, and cluster analysis on topics, Citespace can detect research hot spots and popularity in different periods through keyword burst, thus analyzing the development trends in the field (19). In our study, we utilized cluster analysis to identify key research areas and directions of ILCs in the brain, intestines, and lungs by clustering keywords and co-cited documents. Modularity Q and mean silhouette were used as important evaluation indicators in cluster analysis. It is commonly accepted that Modularity Q > 0.3 indicates a significant clustering structure, while Mean silhouette > 0.5 suggests convincing clustering results. Furthermore, we conducted burst analysis and timeline analysis of keywords, references, and more to explore the hot spots and development trends of ILCs in the fields of brain, intestine, and lung.

When conducting visual analysis using Citespace, we typically analyze the data with the system’s default parameters: time slice=1, link strength measured by cosine, g-index K=25, and top N=50. For clustering, we employ the LLR algorithm. The algorithm used for burst analysis is based on a conference paper titled ‘Bursty and Hierarchical Structure in Streams’ by Kleinberg J in 2002. The number of nodes within a cluster indicates the level of activity in a field, representing an emerging trend in research. During burst analysis, we also follow the system’s default parameters, which involve configuring the detection model with F(x)=ae^-ax^, a_1_/a_0_ = 2.0, a_i_/a_i-1_ = 2.0, The Number of States=2, γ [0,1]=1.0, Minimum Duration=2.

## 3 Results

### 3.1 The trends of annual publication

**Figure 2:**
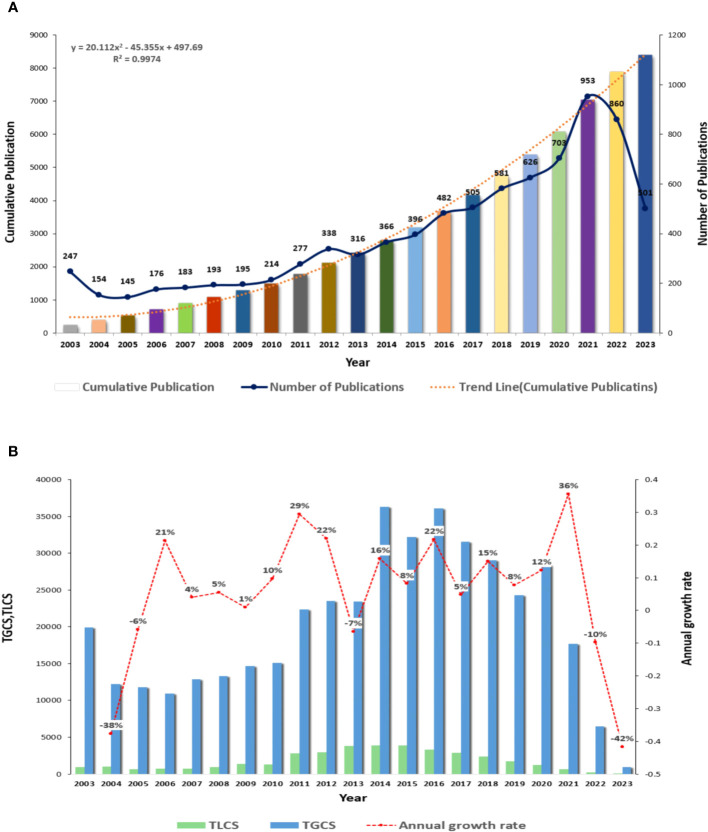
Overall distribution of publications and citations in this field **(A)** Global annual output trends. **(B)** TGCS, TLCS and growth rate in annual publications.

We conducted a comprehensive search of academic articles and reviews on ILCs in the fields of brain, intestine, and lung using Web of Science. A total of 8411 articles were screened, including 6070 articles and 2341 reviews. During the search period, Lin et al. published the first article in this field, which revealed that the expression levels of ICAM-1 and L-selectin on peripheral blood NK cells decreased during acute attacks of childhood asthma. This reduction promoted the migration of NK cells to the lung inflammation site (20). NK cells, the first ILCs discovered in the 1970s, have been the subject of extensive research due to their crucial role in both innate and adaptive immunity. **Figure 2A** illustrates a fitting curve that demonstrates an overall upward trend in the number of publications on ILCs in the brain, intestine, and lung fields since 2003. The average number of publications is approximately 400, with the highest number recorded in 2021 (n=953). In **Figure 2B**, it is evident that the growth rate of published articles in 2020-2021 was the highest at 36%, while the number of published articles declined in the past two years. Over the past 20 years, the total number of local citations for these articles reached 36672, with a global citation count of 417918. The peak values for total local citations (TLCS) and total global citations (TGCS) were observed in 2014 (TLCS=4110, TGCS=36160). However, starting from 2021, there has been a significant decrease in TLCS and TGCS compared to the previous year, reaching the lowest point in 2023 (TGCS=631).

### 3.2 National and regional studies

**Figure 3:**
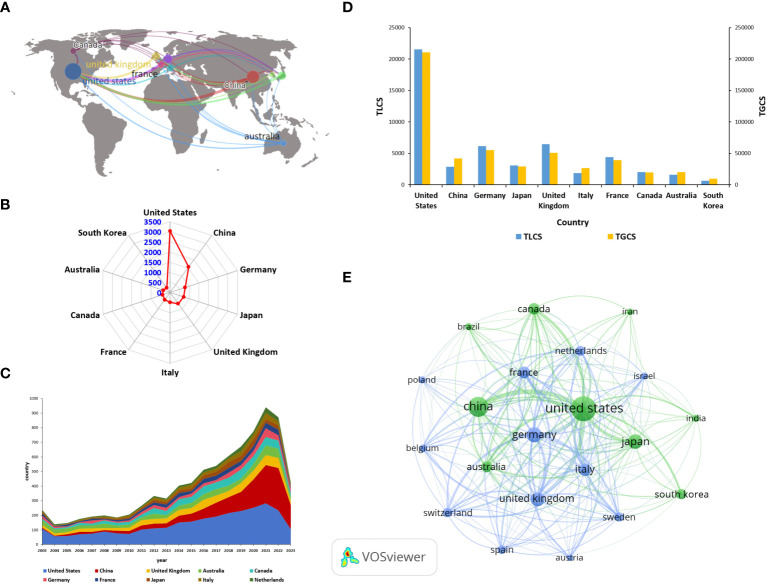
Major countries contributing in this field **(A)** Geographical distribution of global output. **(B)** Radar map of the top 10 productive countries. **(C)** Annual output trend of the top 10 productive countries (The areas of different colors represent the output in different countries). **(D)** Bar chart of TGCS, TLCS of the top 10 productive countries. **(E)** Visual cluster analysis of cooperation among countries (The size of the circle is proportional to the number of articles, and the thickness of the line indicates the strength of the link).

From 2003 to 2023, a total of 99 countries have published articles on ILCs in the fields of brain, intestine, and lung. The geographical distribution of these countries is shown in **Figure 3A**. **Table 1** displays the top ten countries with the most publications. According to **Figure 3B**, the United States has the highest number of publications, with a total of 3044 articles. China (including Taiwan) follows with 1559 articles, and Germany ranks third with 788 articles. In terms of citations, the United States leads with a total of 210776 citations. Germany comes second with 55098 citations, followed by the United Kingdom with 50887 citations. China ranks fourth, as shown in **Figure 3D**. Israel has the highest average number of citations, with 115 publications, 11390 citations, and an average of 99.00 citations per article. France follows with 443 publications, 39235 citations, and an average of 88.57 citations per article. China’s average number of citations is 26.86. **Figure 3E** visually displays the national cooperation relationships in the research on ILCs in the fields of brain, intestine, and lung. Notably, there is strong international research cooperation between China and the United States, followed by the United States and Germany, the United Kingdom, Japan, Canada, and other countries. The annual publication trend chart by country (**Figure 3C**) reveals that research in the United States, the United Kingdom, Australia, and other countries began earlier compared to China. However, the number of publications in China has been steadily increasing since 2010, and in recent years, China has become the leading country in terms of publication output in this field.

**Table 1:** The top 10 most productive countries.

Rank	Country	publications	TLCS	TGCS	Average Citation	Centrality
1	United States	3044	21536	210776	69.24	0.21
2	China	1559	2868	41875	26.86	0.05
3	Germany	788	6135	55098	69.92	0.13
4	Japan	708	3061	29074	41.06	0.09
5	United Kingdom	681	6477	50887	74.72	0.15
6	Italy	483	1862	26419	54.70	0.06
7	France	443	4391	39235	88.57	0.28
8	Canada	381	2039	19882	52.18	0.04
9	Australia	363	1599	20447	56.33	0.05
10	South Korea	283	634	9810	34.66	0.00

### 3.3 Analysis of research institutions and study authors

**Figure 4:**
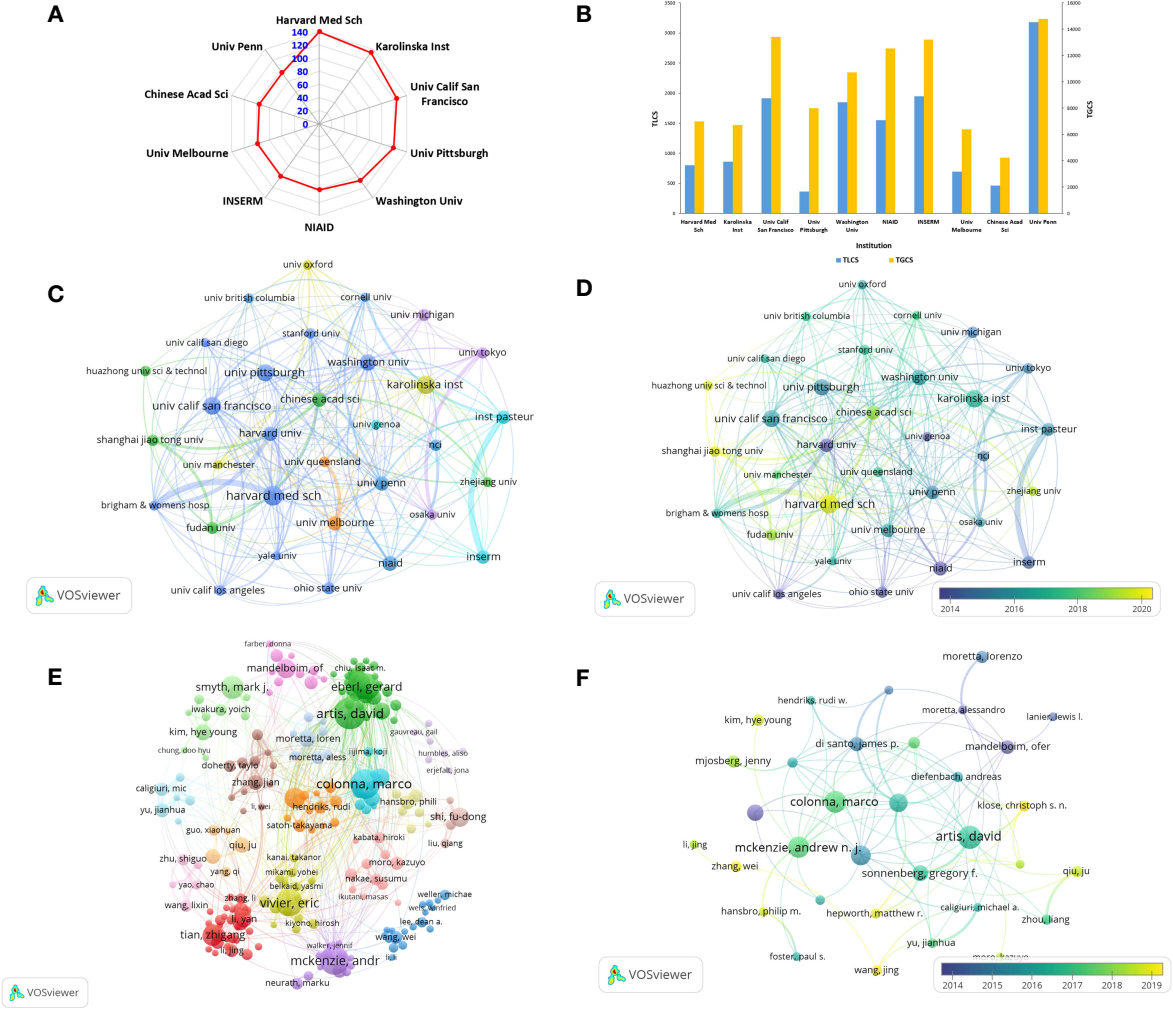
Contributions and relationships with major research institutions or authors **(A)** The top10 institutions with the number of publications. **(B)** The number of citations corresponding to the top 10 institutions. **(C)** Visual cluster analysis of cooperation among institutions. **(D)** Timeline visualization of cooperation among institutions (different colors represent different times). **(E)** Visual cluster analysis of cooperation among authors (different colors represent different clusters). **(F)** Timeline visualization of cooperation among authors.

A total of 45279 authors from 6585 institutions participated in the research on ILCs in the fields of brain, intestine, and lung. The top 10 institutions with the most research results are presented in **Figure 4A**. Harvard Medical School in the United States leads with the highest number of publications (n=140, TGCS =6997, TLCS=800), followed by Karolinska Institutet in Sweden (n=134, TGCS=6709, TLCS=860), and University of California in the United States (n=124, TGCS=13404, TLCS=1914). In terms of total global citations (TGCS), the University of Pennsylvania in the United States holds the top position with 14,778 citations, followed by the University of California in the United States (TGCS=13404) and Inserm in France (TGCS=13200) (**Figure 4B**). The social network diagram of research institutions illustrates the division of institutions into 7 clusters based on an occurrence frequency of ≥10 and cooperative relationships (**Figure 4C**). Notably, there is significant collaboration between institutions, both domestically and internationally, with the strongest partnership observed between France’s Inst Pasteur and Inserm. According to **Figure 4D**, articles published by Harvard University and NIAID in the United States were published earlier. In contrast, Chinese institutions such as Shanghai Jiao Tong University and Huazhong University of Science and Technology have published a higher number of articles around 2020.

The top three authors with the most published articles on innate lymphoid cells (ILCs) in the fields of brain, intestines, and lungs are David Artis from the University of Pennsylvania (n=40, TGCS=10327, TLCS=2857), Marco Colonna from Washington University (n=37, TGCS=6803, TLCS=1510), and Andrew NJ McKenzie from the MRC Laboratory of Molecular Biology (n=36, TGCS=6866, TLCS=2158) (**Table 2**). David Artis is highly regarded as an authoritative expert in the fields of brain, intestines, and lungs, as evidenced by his extensive research publications on ILCs. His articles have received the highest number of citations in this field. Notably, he published the first review on this topic in 2010 and has since continued his uninterrupted research on ILCs. In October 2023, David Artis published a comprehensive review in the journal Immunology, providing an in-depth analysis of ILC2s and TH2 cells and their synergistic effect in the expulsion of intestinal parasites (21). After conducting visualization (**Figure 4E**), we observed a relatively close cooperation among the authors. We formed 18 clusters consisting of authors who had an occurrence frequency of ≥10 and a cooperative relationship. The strongest collaboration was found between Colonna and Gilfillan. In terms of publication timing, most authors published their papers after 2015, as depicted in **Figure 4F**.

**Table 2:** The top 10 most productive authors.

Rank	Name	Country	Counts	TLCS	TGCS	H-index
1	Artis D	United States	40	2857	10327	86
2	Colonna M	United States	37	1510	6803	140
3	McKenzie ANJ	United Kingdom	36	2158	6866	79
4	Vivier E	France	34	1256	6579	105
5	Eberl G	France	32	1824	7891	68
6	Smyth MJ	Australia	28	223	4657	145
7	Sonnenberg GF	United States	27	1321	4203	36
8	Di Santo JP	France	25	1258	6491	87
9	Tian Z	China	25	225	1763	77
10	Shi F	China	23	286	1551	54

### 3.4 Journal analysis

**Figure 5:**
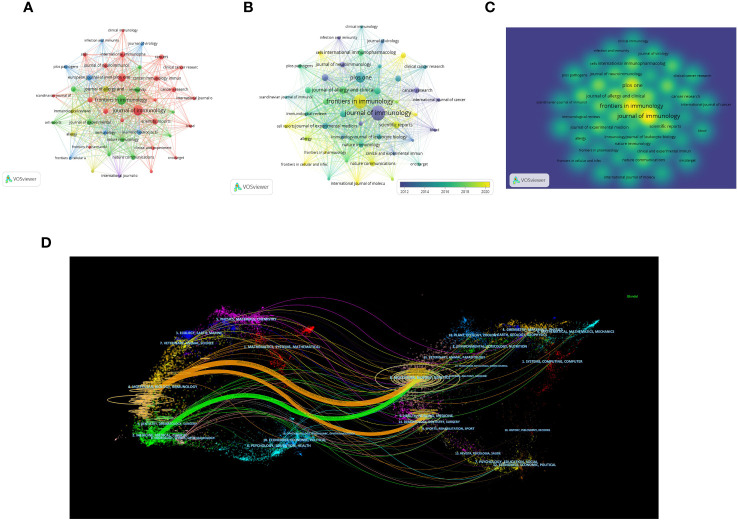
Distribution of the main journals publishing research in the field **(A)** Visual cluster analysis among Journals. **(B)** Timeline visualization among Journals. **(C)** Density visualization of journals (The color brightness is directly proportional to the density of the publication). **(D)** The dual-map overlap of journals (The left side represents the citing journal, the right side represents the cited journal, and the line path represents the citation relationship).

All articles were published in 1350 magazines. The top 10 magazines with the most articles are shown in **Table 3**. According to **Figure 5A**, The Journal of Immunology leads with 330 articles (n=330, TGCS=20331, TLCS=2247), followed by Frontiers in Immunology with 289 articles (n=289, TGCS=9135, TLCS=2), and Plos One with 199 articles (n=199, TGCS=6294, TLCS=0). However, these journals have relatively low citation rates in the field. The top three journals with the highest citation counts are Immunity (TGCS=22719), Journal of Immunology (TGCS=20331), and Nature Immunology (TGCS=17537). **Figures 5B, C** depict the journal time series diagram and heat map, respectively. Notably, Frontiers in Immunology has gained popularity for submissions in recent years. According to the double overlay diagram, there are three main citation paths. The published articles were predominantly found in journals related to molecular biology, immunology, and clinical medicine. On the other hand, the cited articles were mostly published in journals covering molecular biology, genetics, health, nursing, and medicine (**Figure 5D**).

**Table 3:** The top 10 most productive journals.

Rank	Journal	Counts	TLCS	TGCS	IF(2023)
1	JOURNAL OF IMMUNOLOGY	330	2247	20331	4.4
2	FRONTIERS IN IMMUNOLOGY	289	2	9135	7.3
3	PLOS ONE	199	0	6294	3.7
4	JOURNAL OF ALLERGY AND CLINICAL IMMUNOLOGY	128	2458	10514	14.2
5	SCIENTIFIC REPORTS	112	0	1911	4.6
6	IMMUNITY	101	4129	22719	32.4
7	EUROPEAN JOURNAL OF IMMUNOLOGY	100	806	5076	5.4
8	INTERNATIONAL IMMUNOPHARMACOLOGY	98	89	1827	5.6
9	CANCER IMMUNOLOGY IMMUNOTHERAPY	93	249	4410	5.8
10	MUCOSAL IMMUNOLOGY	92	943	4788	8.0

### 3.5 Literature co-citation analysis

**Figure 6:**
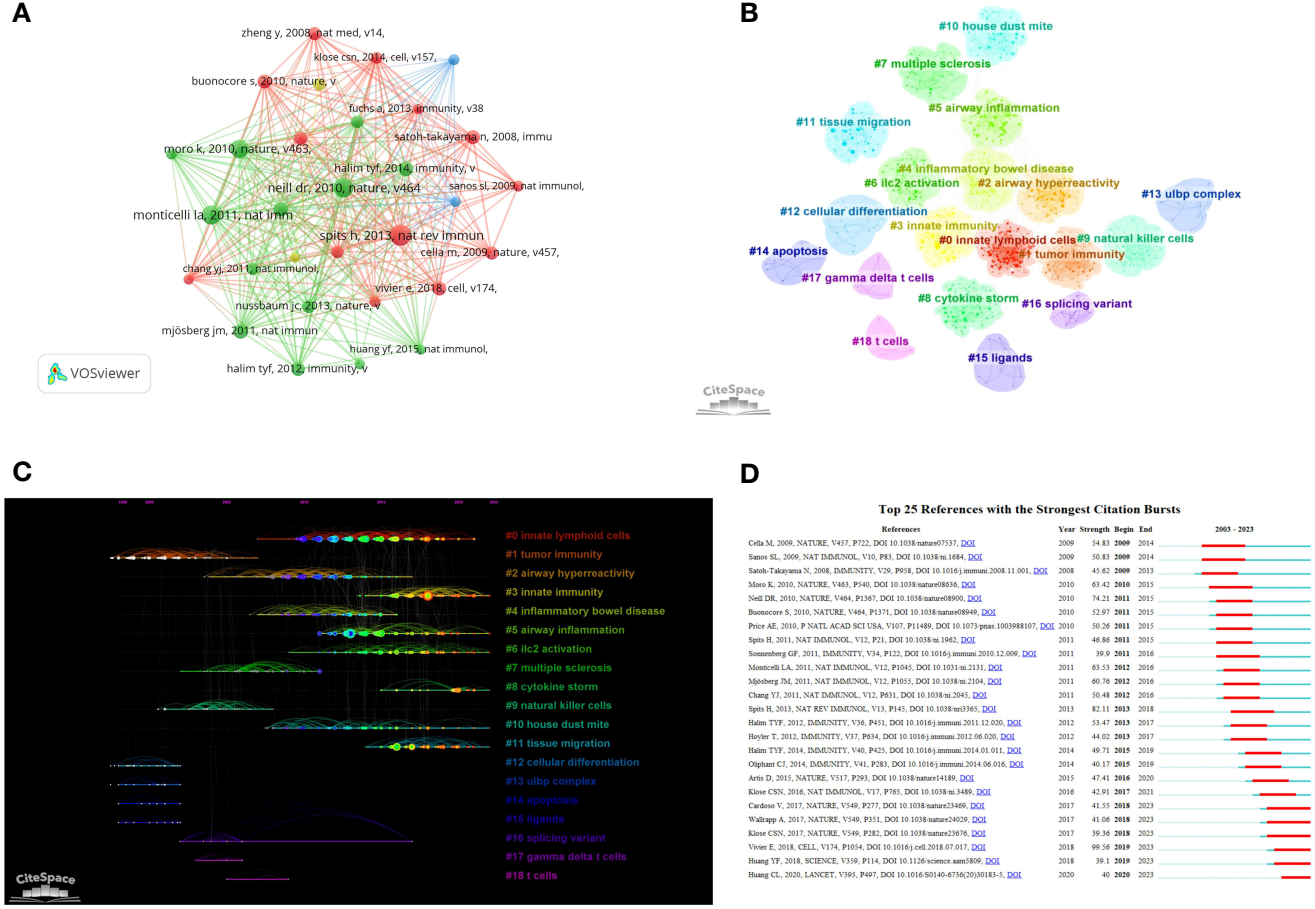
Clustering and bursting of co-cited references **(A)** Visual analysis of Co-cited References. **(B)** Cluster Analysis of Co-cited References. **(C)** Timeline distribution of the 19 clusters (The length of the lit line indicates the start and end time of the hotspot. The further back the line is, the closer it is to the present moment). **(D)** The top 25 references with the strongest citation bursts (The length of the red line indicates the duration of the hotspot).

**Table 4** presents the top 10 most frequently cited articles. These articles provide comprehensive descriptions of the roles and mechanisms of various types of ILCs in inflammation, allergy, and infection. By conducting a co-citation analysis of the 278,967 references, it was discovered that there were 139 articles with more than 100 citations. The article with the highest number of citations, authored by Hergen Spits et al. and published in the journal Nature reviews Immunology in 2013. In their review, the authors proposed a classification of ILCs into three categories based on the cytokines produced by ILCs and the transcription factors that regulate their development and function. This classification has been widely accepted and is now considered the standard for categorizing ILCs. It has significantly advanced research on ILCs in the field of brain-gut-lung axis (3). We constructed a network visualization of co-cited documents (**Figure 6A**) and conducted cluster analysis, which led to the identification of 19 cluster modules (**Figure 6B**). The modularity Q value was determined to be 0.8900, and the mean silhouette value was 0.9055. These cluster modules encompassed various topics such as Innate lymphoid cells, Tumor immunity, Airway inflammation, Inflammatory bowel disease, Cytokine storm, and ILC2 activation. Additionally, we visualized the clustering timeline (**Figure 6C**) and observed frequent mentions of Tumor immunity, NK cells, Multiple sclerosis, Inflammatory bowel disease, Ligands, Airway hyperreactivity, etc. in the early research on ILCs in the brain, intestine, and lung fields. The inclusion of these references suggests that they are considered research hotspots in this particular domain. The current focus in this research field is on Innate lymphoid cells, Immunity, Airway inflammation, Cytokine storm, and Tissue migration. Upon closer examination of the clustering patterns, it was observed that ‘#0 Innate lymphoid cells’ mainly refers to ILC1, ILC2, and ILC3. These three types of cells have been progressively recognized since 2010. Notably, in 2010, *Sawa* et al. proposed in Science that RORγt(+) innate lymphocytes (ILC) play a beneficial role in maintaining intestinal bacteria, stabilizing the population, and promoting the production of intestinal defense cells (22). This groundbreaking study paved the way for further research on these three types of ILCs in various organs such as the brain, intestine, and lungs. An burst analysis was conducted on the co-cited documents (**Figure 6D**). The review by Vivier et al. in 2018 (4) had the highest burst strength. This review provided detailed information on the research conducted in the past ten years, emphasizing the importance of ILCs in environmental homeostasis, tissue metabolism, and cell repair. Currently, the focus of the cited literature is on an article published by Huang et al. in Nature in 2018. They discovered that interleukin-25 or helminth-induced inflammation ILC2s can facilitate the chemotaxis of sphingosine 1-phosphate (S1P), enabling migration from the intestinal lamina propria to various tissues through blood and lymph circulation (12). In 2017, Cardoso et al. found that neuromodulation of type 2 innate lymphocytes can be achieved through neuromedin U (23). Additionally, Klose et al (24). published an article in Nature in the same year, suggesting that the nervous system regulates ILCs and type 2 inflammatory cells through the neuropeptide neuromedin U to mediate intestinal immune protection. This research area has gained considerable attention, highlighting the significance of neural regulation in intestinal immunity in recent years.

**Table 4:** The top 10 most cited references.

Rank	First author	Journal	DOI	Year	IF(2023)
1	Spits H	NAT REV IMMUNOL	DOI 10.1038/nri3365	2013	100.3
2	Neill DR	NATURE	DOI 10.1038/nature08900	2010	64.8
3	Monticelli LA	NAT IMMUNOL	DOI 10.1031/ni.2131	2011	30.5
4	Mjösberg JM	NAT IMMUNOL	DOI 10.1038/ni.2104	2011	30.5
5	Price AE	PNAS	DOI 10.1073/pnas.1003988107	2010	9.58
6	Halim TYF	IMMUNITY	DOI 10.1016/j.immuni.2014.01.011	2014	32.4
7	Vivier E	CELL	DOI 10.1016/j.cell.2018.07.017	2018	64.5
8	Satoh-Takayama N	IMMUNITY	DOI 10.1016/j.immuni.2008.11.001	2008	32.4
9	Buonocore S	NATURE	DOI 10.1038/nature08949	2010	64.8
10	Artis D	NATURE	DOI 10.1038/nature14189	2015	64.8

### 3.6 Keyword hot spots and word frequency analysis

**Figure 7:**
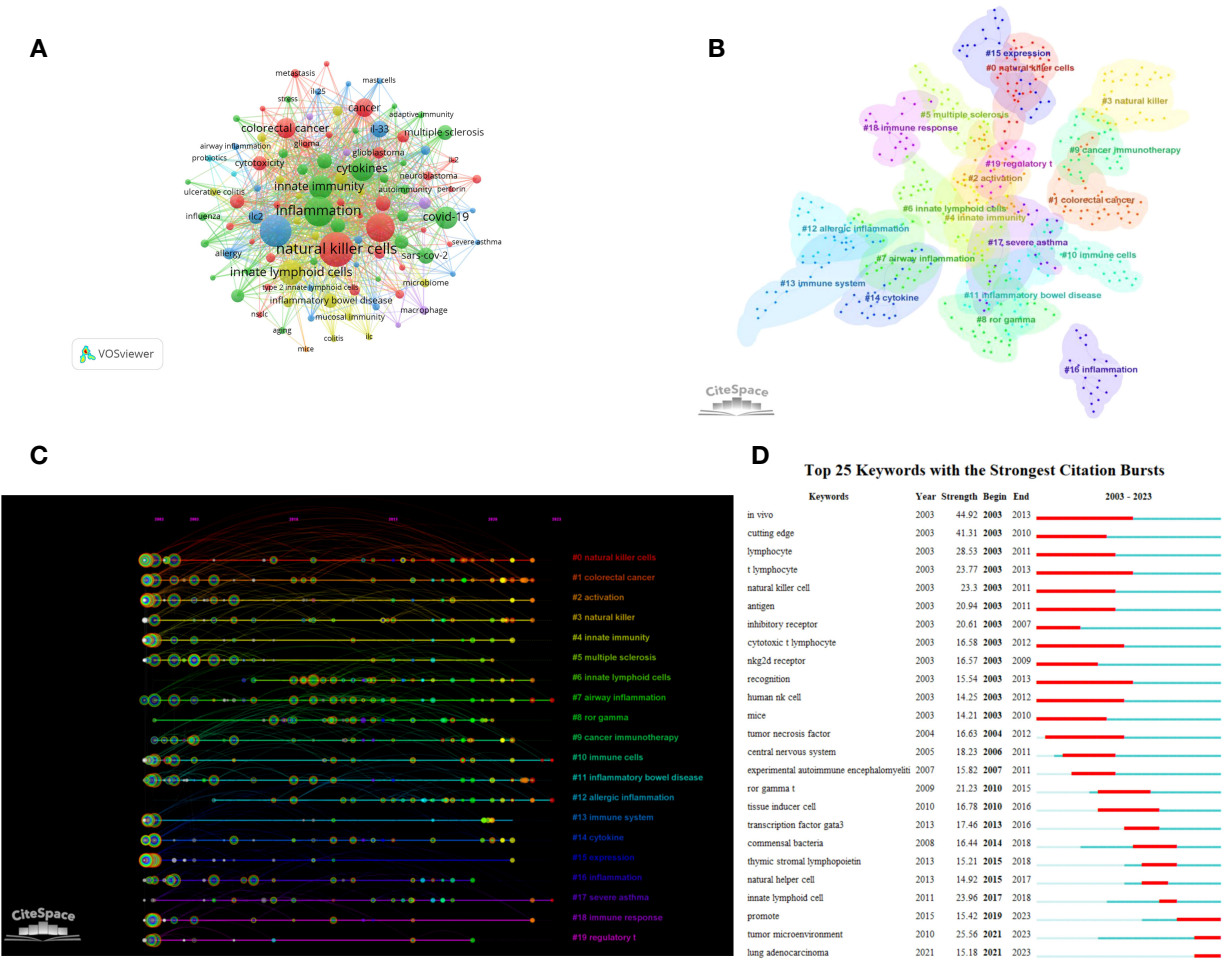
Clustering and bursting of keywords **(A)** The network map of keywords. **(B)** Cluster Analysis of keywords. **(C)** Timeline distribution of cluster analysis of keywords. **(D)** The top 25 keywords with the strongest citation bursts.

A visual analysis was conducted on 21,439 keywords in this field (**Figure 7A**). Keywords such as ILCs, NK cells, inflammation, asthma, cancer, dendritic cell, and immunotherapy appeared frequently in this field, with a frequency of more than 500 times. The cluster analysis resulted in 20 clusters (**Figure 7B**) with a modularity Q of 0.816 and a mean silhouette value of 0.9363. Combining the clustering timeline results, **Figure 7C** demonstrates that cancer, immunity, airway inflammation, asthma, RORγT, immunotherapy, inflammatory bowel disease, allergic inflammation, and other related topics have been the primary areas of focus in the research on ILCs in the brain, intestine, and lung since 2003. These findings highlight the key research topics in this field. We conducted an burst analysis on keywords and identified the top 25 burst keywords. **Figure 7D** displays the keyword with the highest burst strength, which is ‘*in vivo*’, and it remains burst for a duration of 10 years. Additionally, our analysis revealed that lung adenocarcinoma and tumor microenvironment are prominent keywords in recent research. This suggests that ILCs may significantly impact the diagnosis and treatment of brain, intestinal, and lung tumors.

After analyzing the keywords and co-cited documents, recent studies have focused on investigating ILCs in the brain, intestine, and lung. These studies primarily explore various aspects including allergic diseases, intestinal inflammation, infection, tumors, and neuroendocrine regulation. Notably, ILCs have been found to play a significant role in the brain-gut axis and the lung-gut axis. However, the specific role of ILCs in the brain-lung axis remains unclear, and there is a lack of research in this area. Understanding the contribution of ILCs in the brain-lung axis is crucial for establishing a comprehensive research framework on the brain-gut-lung axis. Therefore, further research is needed to fill the existing gaps and advance our knowledge in this domain.

### 3.7 ILCs and tumors (brain, intestine and lung fields)

**Figure 8:**
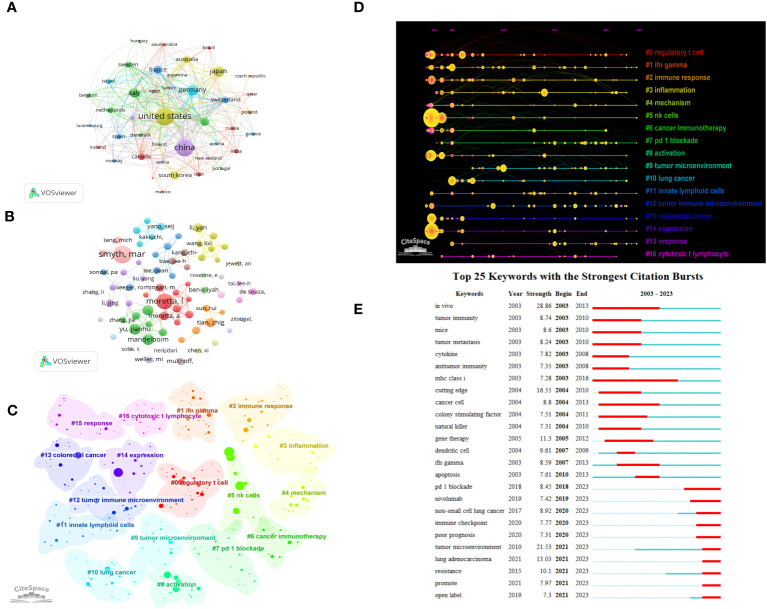
Visualization of oncology-related articles in the field **(A)** Visual cluster analysis of cooperation among countries. **(B)** Visual cluster analysis of cooperation among authors. **(C)** Cluster Analysis of keywords. **(D)** Timeline distribution of cluster analysis of keywords. **(E)** The top 25 keywords with the strongest citation bursts.

Based on the above search terms, we added the conditions “Tumor” OR “Neoplasm” OR “Neoplasia” OR”Cancer” OR “Malignan”, A total of 5597 articles were retrieved, and after screening, 2709 articles related to ILCs in brain, intestinal, and lung tumors were included. In terms of publications, the United States leads with the highest number (n=821), followed by China (n=740) and Japan (n=252) (**Figure 8A**). The United States is also the most cited country (TGCS=53717), followed by China (TGCS=18282). The most cited article is a 2009 article by *Castriconi* et al., they discovered that NK cells have the ability to identify and eliminate human glioblastoma cells that exhibit stem cell-like characteristics (25). Moving on to authors, Smyth has the highest number of publications in this field (n=23) (**Figure 8B**). Keyword clustering of the literature resulted in 17 clusters (Q=0.7532, S=0.8871) as shown in **Figure 8C**, Among the various types of ILCs, NK cells have received the most attention in research. Time visualization and burst analysis of the clusters reveal that recent research has focused on hot topics like IFN-gamma, mechanism, immunotherapy, tumor microenvironment, immune checkpoint, lung cancer, colorectal cancer, and PD-1 (**Figures 8D, E**). PD-1 is a crucial immunosuppressive molecule (26, 27). The combination of PD-1 and PD-L1 reduces T cell activity and suppresses the immune system’s attack on tumor cells. Blocking the PD-1 and PD-L1 combination restores T cell activity, leading to effective tumor cell killing and remarkable efficacy in various cancer treatments. PD-1/PD-L1 monoclonal antibodies are currently the most extensively studied immune checkpoint inhibitors in clinical research and application, making them a prominent topic in tumor immunotherapy in recent years.

## 4 Discussion

We conducted an analysis of 8411 documents that were published by 45279 authors affiliated with 6585 institutions across 99 countries. Over time, the annual publication volume has been consistently increasing. While there has been a slight decrease in the number of citations for articles in the past two years, there is still significant attention and understanding in this field. The United States stands out as the country with the highest number of publications and the highest TGCS, indicating its significant influence in this field. One noteworthy contributor is David Artis from the University of Pennsylvania, who has made substantial contributions to the study of the role of ILCs in immune regulation and tissue homeostasis. His research focuses on the relationship between ILCs and various aspects such as the intestines, lungs, skin, mucosal barriers, and obesity (1, 28). Recently, he has shifted his focus towards understanding the non-redundant functions of ILC2 in the lungs and intestines. He has proposed that neuropeptides, specifically a cholinergic neuropeptide, regulate the anti-parasitic and protective effects of ILC2 through neurons, thereby establishing a connection between the lung-gut axis and neurotransmitters. This advancement in research sheds light on the brain-gut-lung axis and provides valuable insights into the interplay between ILCs and this axis (29, 30). According to **Figure 3C**, it is evident that developed countries such as the United States, Britain, and Canada have been researching in this field for a longer period, while China started its research later. However, China has maintained close collaboration with European and American countries, resulting in a significant increase in the number of publications in recent years.

Among all the journals to which articles were submitted, Immunity, Journal of Immunology, and Nature Immunology are the top three journals with the highest number of citations. They have a larger number of publications compared to traditional top journals such as Nature, Science, Cell, and Lancet. This suggests that they are esteemed journals in the field and are preferred by many scholars studying the relationship between ILCs and the brain, intestines, and lungs. This preference is due to the fact that the research scope of ILCs is related to immunity, and Nature and Science are comprehensive journals. In Immunity magazine, the highest TLCS was published by Halim et al. in 2014: ‘Group 2 innate lymphoid cells are critical for the initiation of adaptive T helper 2 cell-mediated allergic lung inflammation’ (31). This publication emphasizes the role of ILC2 in asthma and other allergic diseases, attracting significant attention from colleagues. Another immunology journal, Frontiers in Immunology, has witnessed a significant increase in the number of articles published in recent years, reaching 7792 articles in 2022-2023. However, its TLCS remains relatively low.

Cluster analysis and timeline chart of co-cited documents and keywords indicate that research on ILCs in the brain, intestine, and lung fields may involve asthma, tumor, Cytokine storm, Multiple sclerosis, Inflammatory bowel disease, immunotherapy, RORγT. Asthma, characterized by airway hyperresponsiveness, is closely linked to ILCs, particularly ILC2, which plays a significant role. Activation of ILC2 by external allergens or cytokines (such as IL-25 and IL-33) leads to the production of type 2 cytokines, resulting in an increasing in eosinophils, mast cells, mucus secretion, and mucosal tissue remodeling. These processes contribute to the development of allergic diseases like asthma (32–34). As a result, researchers have been focused on studying the excessive activation of ILC2 and the associated type 2 inflammatory responses, aiming to identify new targets for asthma treatment. Multiple sclerosis (MS) is a well-known autoimmune disease that affects the central nervous system. Numerous studies have demonstrated the significant involvement of ILCs in both the onset and progression of this disease (35, 36). The interaction between the nervous system and immune cells, such as ILCs, is complex and not fully understood (37). The nervous system, including the central nervous system, plays a crucial role in regulating the immune response of tissues such as the intestines and lungs, maintaining the body’s physiological homeostasis (5). In a study by Ibiza et al. (38), they discovered that glial cells promote the activation of RET tyrosine kinase by regulating the expression of neurotrophic factors. This activation induces intestinal ILC3 to produce a significant amount of IL-22, which helps maintain intestinal stability, inhibits inflammation, and prevents infection. Neuromedin U (NMU) is a highly conserved sequence that plays a crucial role in activating ILC2s in intestinal and lung tissues. Cholinergic neurons produce NMU, which promotes the production of types 2 inflammation (24). NMU activation leads to the activation of eosinophils and mast cells, resulting in smooth muscle contraction and contributing to the development of allergic diseases (39, 40). Therefore, NMU is vital for maintaining the homeostasis of ILC2s and overall body balance. These findings provide evidence of the crucial role of the nervous system in regulating ILCs and maintaining the stability of the intestine and lungs. This lays the foundation for further exploration of the brain-gut-lung axis and the role of ILCs. The transcription factor RORγT is responsible for regulating the expression and development of ILC3 (41, 42). It promotes the production of inflammatory factors IL-22 and IL-17, leading to increased goblet cell mucin secretion and inducing intestinal epithelial fucosylation. These effects enhance the integrity of the intestinal barrier and help maintain the homeostasis of the intestinal flora. However, some studies have shown (43, 44) that ILCs can respond to IL-23-mediated inflammatory bowel disease (IBD) by producing IL-22 and IL-17. This has led to an increasing focus on the role of ILCs in IBD, with researchers striving to discover new targets for its treatment (45, 46).

With the advancement of research on ILCs in the brain, intestine, and lung, new research directions in this field have gained prominence. The investigation of ILCs in brain tumors, intestinal tumors, and lung tumors has become a major area of interest in recent years, attracting significant attention from researchers. Particularly, keywords such as monoclonal antibodies and PD-1 have garnered significant interest. However, the precise mechanism of action of ILCs in tumors remains unclear. ILCs exhibit dual characteristics, resembling a politician with affiliations to both parties (47–49). This is because the function of ILCs is influenced by various factors, including phenotypic subgroups, signaling pathways, tissue sites, and the tumor microenvironment. These factors contribute to the divergent effects of ILCs in tumor development. NK cells, the first type of ILCs to be discovered, are known for their high plasticity (50). In the context of tumors, NK cells can exhibit both pro-tumor and anti-tumor phenotypes. They possess the ability to inhibit tumor growth and metastasis (51), thereby extending the lifespan of patients. While studies have highlighted the improved prognosis and overall survival rate associated with NK cells (52), there are also concerns about their potential carcinogenic risk (53). Apart from NK cells, researchers have shown significant interest in the other three types of ILCs - ILC1, ILC2, and ILC3. In a study by Saranchova et al. (54, 55), it was reported that IL-33 can alter the tumor microenvironment, leading to the activation of anti-tumor CD8+ T cells and eosinophils by ILC2. This activation inhibits tumor formation and suppresses lung tumor metastasis, with the ILC2-IL-5-GM-CSF axis playing a crucial role in this process (47). According to research conducted by Jacquelot and others, IL-33 has the ability to enhance tumor sensitivity to PD-1 by activating ILC2. This activation results in an improved anti-tumor effect of PD-1, leading to positive outcomes for the majority of patients (56, 57). However, Schuijs et al. discovered in their study that ILC2s activated by IL-33 can inhibit the tumor-killing effect of NK cells, leading to tumor metastasis (58). Jou et al. also found in their research (59) that IL-25 can activate ILC2 and recruit myeloid-derived suppressor cells (MDSC) in colorectal tumors. This activation inhibits the function of anti-tumor CD8+ T cells and promotes tumor development. Both NK cells and ILC1s can eliminate tumor cells by secreting granzyme, perforin, and IFN-γ. Under certain conditions, NK cells can differentiate into ILC1s, with NK cells primarily inhibiting tumor growth and ILC1s more effectively inhibiting tumor metastasis (60, 61). Additionally, ILC3s accumulate in the meninges, where they promote anti-tumor immunity in brain cancer by facilitating T lymphocyte infiltration into the brain and supporting their survival (62).

This study has a few limitations that need to be acknowledged. Firstly, our study is primarily focused on examining the function and role of ILCs in the brain, intestine, and lung. Although we made efforts to enhance our search terms and strategies, it is still possible that we may have missed some relevant studies. Secondly, our data is solely obtained from the WOSCC database, which means our analysis is restricted to English articles and reviews. Lastly, it is important to mention that a substantial portion of our results depend on machine algorithms, with limited manual induction and organization.

## 5 Conclusion

Currently, there is a growing research interest in the field of innate lymphoid cells (ILCs) in the brain, intestine, and lung. In this study, we conducted a bibliometric analysis to identify research trends and hot spots in this field. Our findings reveal that asthma, tumor, Cytokine storm, Multiple sclerosis, Inflammatory bowel disease, immunotherapy, RORγT, neuroimmunology, and PD-1 are the primary focus and hot spots of ILC research in the brain, intestine, and lung. Among the various types of ILCs, NK cells have received the most attention in research. Researchers have dedicated significant efforts to these areas. However, most studies tend to concentrate on a single system or organ, or the brain-gut and lung-gut axes. The relationship and regulation between ILCs in the brain-lung axis remain unclear. It is essential for researchers to investigate the role of ILCs in the brain-lung axis, the specific mechanisms underlying related diseases, and the reasons behind the high metastasis of lung cancer to the brain. These questions warrant further exploration.

## Data availability statement

All datasets presented in this study can be found in the article and WoSCC database.

## Author contributions

JH: Writing – original draft, Formal Analysis, Visualization. KD: Formal Analysis, Visualization, Writing – original draft. YL: Formal Analysis, Visualization, Writing – original draft. MX: Formal Analysis, Visualization, Writing – original draft. ML: Formal Analysis, Visualization, Writing – original draft, Writing – review & editing. MW: Formal Analysis, Visualization, Writing – original draft, Writing – review & editing.
